# Death by Violence

**Published:** 1893-01

**Authors:** 


### DEATH BY VIOLENCE.

Most people regard death by a fall as one of the most agonizing forms of dying. In a lecture at Zurich, Professor Heim has declared, says a Berlin correspondent, that this opinion is erroneous. The first fact to be considered, according to the professor, is that the subjective feelings in the various kinds of fall are the same. There are people who have escaped death by a hair's breadth, who reached the stage of unconsciousness, and who are able to report what they felt. Professor Heim, who has occupied himself with this interesting question for many years, bases his observations on personal experiences, and on a large number of cases which have occurred, not only in the mountains, but also in war, in industrial establishments and in railway accidents. The victim suffers no pain, no paralyzing terror. He is perfectly aware of what is going on. The time seems long to him. In a few seconds he is able to think so much that he can report an entire hour on it. His thinking power is immensely increased. In almost all cases the past seems suddenly lighted up as if by a flash of lightning. All phases of life pass before the mind's eye, nothing petty or unimportant disturbing the retrospect. Then gentle, soft tones sound in one's ears and die away at last when unconsciousness sets in. One hears the fall of the body, but one does not feel it. It will be remembered that Mr. Whymper, who had a severe succession of falls once in the Alps without losing his consciousness, declares emphatically that as he bounded from one rock to another he felt absolutely no pain. The same thing happens on the battle field; the entrance of the bullet into the body is not felt, and it is not till he feels the blood flowing or a limb paralyzed that the soldier knows he is wounded. Persons who have had limbs broken by a fall do not know which limbs are affected till they try to rise. At the moment of a fall the whole intellectual activity is increased to an extraordinary degree. There is not a trace of anxiety. One considers quickly what will happen or may happen. This is by no means the consequence of presence of mind, it is rather the product of absolute necessity. A solemn composure takes possession of the victim. Death by fall is a beautiful one. Great thoughts fill the victim's soul; they fall painlessly into a great blue sky.

